# Pancreatic cancer burden in China from 1990 to 2023: an analysis based on the global burden of disease study 2023

**DOI:** 10.3389/fendo.2026.1884013

**Published:** 2026-07-15

**Authors:** Ziyue Wang, Shuai Wang, Guomu Liu, Hao Wu, Yuguo Chen

**Affiliations:** 1 Department of Hepatobiliary and Pancreatic Surgery, General Surgery Center, The First Hospital of Jilin University, Changchun, China; 2 Key Laboratory of Organ Regeneration and Transplantation of Ministry of Education, and National-Local Joint Engineering Laboratory of Animal Models for Human Diseases, The First Hospital of Jilin University, Changchun, China; 3 Department of Gastric and Colorectal Surgery, General Surgery Center, The First Hospital of Jilin University, Changchun, Jilin, China; 4 Department of Dermatology and Venereology, The First Hospital of Jilin University, Changchun, Jilin, China; 5 Department of Urology, The First Hospital of Jilin University, Changchun, China

**Keywords:** age-specific trends, China, global burden of disease, joinpoint, pancreatic cancer

## Abstract

**Background:**

Pancreatic cancer (PC) poses a significant public health challenge in China because of high mortality and increasing incidence, necessitating an analysis of its burden from 1990 to 2023 to inform future prevention.

**Methods:**

Using data from the Global Burden of Diseases, Injuries, and Risk Factors Study (GBD) 2023, we estimated absolute numbers, age-standardized rates (ASRs), and age-stratified crude rates. Joinpoint regression was employed to calculate average annual percentage changes (AAPCs). We quantified the burden attributable to specific risk factors and conducted a segmented analysis to compare trends between the pre-pandemic and pandemic periods. All estimates are presented with 95% uncertainty interval (UI).

**Results:**

In 2023, China was estimated to have 118,381 incident PC cases (95% UI 103,773-135,339), 114,949 deaths (95% UI 101,214-131,619), 102,845 prevalent cases (95% UI 89,758-117,757), and 2,765,626 DALYs (95% UI 2,455,494-3,137,032). The age-standardized incidence rate (ASIR), age-standardized mortality rate (ASMR), and age-standardized disability-adjusted life year rate (ASDR) remained stable. The age-standardized prevalence rate (ASPR) increased slightly (AAPC 0.38%, 95% CI 0.06 to 0.70). The burden was consistently higher in males than in females. Age-specific analysis highlighted rising incidence rates in both 15–49 years and ≥75 years age groups. In 2023, high fasting plasma glucose (HFPG) accounted for a higher attributable burden than smoking. Pandemic-era sensitivity analysis suggested a possible change in the direction of ASIR and ASMR trends after 2019, but the observed post-2019 increases were not statistically significant and require further validation.

**Conclusion:**

The increasing absolute burden was mainly associated with population aging, while risk-attributable analysis suggested a growing contribution of metabolic factors. Prevention strategies should integrate metabolic management to tackle the dual challenges of an aging society and emerging risks in younger people.

## Introduction

Pancreatic cancer is one of the most fatal malignancies and imposes a substantial public health burden worldwide (1, 2). In China, the cancer burden and the patterns of risk exposure have been changed by population aging, urbanization, and shifts in diet and lifestyle over recent decades (3–6). Updated estimates of pancreatic cancer burden are needed to guide prevention priorities, health resource allocation, and clinical decision making. Previous studies have examined the burden of pancreatic cancer in China, but most relied on earlier GBD estimates and may not fully reflect recent changes in population structure and risk exposure (7, 8). Rapid aging, urbanization, and metabolic transition in China may have altered the scale of the pancreatic cancer burden and the relative importance of modifiable risk factors (9, 10). These changes could raise the absolute burden even when ASRs change little. They could also shift the underlying risk profile. The COVID-19 pandemic, which disrupted cancer diagnosis and treatment, may have affected short-term trends, but such effects are uncertain and require cautious interpretation (11, 12).

We assessed the burden of pancreatic cancer in China from 1990 to 2023 using the most recent GBD 2023 data. The indicators included incidence, mortality, prevalence, and disability-adjusted life years (DALYs). Compared with previous GBD-based studies, this analysis extends the observation period to 2023 and uses updated GBD risk-attributable estimates. We further examined sex- and age-specific trends, decomposed the drivers of burden changes, and explored short-term trend patterns before and after 2019.

## Methods

### Data source and study design

We analyzed data from the Global Burden of Disease Study (GBD) 2023, coordinated by the Institute for Health Metrics and Evaluation, to examine the longitudinal burden of pancreatic cancer in China (1990–2023). By synthesizing diverse data sources, including vital registration, cancer registries, and censuses, through standardized modeling, the GBD framework provides comparable estimates of health loss across 204 countries and territories (13, 14). The extracted indicators included incident cases, deaths, prevalent cases, DALYs, ASRs, age-specific rates, and risk-attributable estimates. All estimates are reported with 95% UI to account for modeling variance.

For this study, pancreatic cancer was defined as malignant neoplasms of the pancreas, classified under ICD-10 code C25 (15).

### Measures of burden

We assessed the disease burden of pancreatic cancer using four core indicators: incidence, mortality, prevalence, and DALYs. DALYs were defined as the sum of years of life lost (YLLs) and years lived with disability (YLDs) (16):


DALYs = YLLs + YLDs

To eliminate age structure differences, we calculated the ASRs per 100,000 population using the GBD global standard population. We also calculated the crude rate for each age group to evaluate the overall burden on the healthcare system.

### Decomposition analysis

We performed a decomposition analysis to quantify the relative contributions of population growth, population aging, and epidemiological change to changes in pancreatic cancer burden between 1990 and 2023. In this analysis, epidemiological change refers to changes in age-specific rates after accounting for population size and age structure (17, 18).

### Risk factor quantification

We used the GBD Comparative Risk Assessment framework to quantify the burden of pancreatic cancer attributable to modifiable risk factors (19). Based on the Global Burden of Disease Study 2023, we focused on four key risk factors: smoking, HFPG, high body mass index (BMI), and high alcohol use. Risk-attributable estimates were obtained from the GBD 2023 Comparative Risk Assessment framework. The burden attributable to each risk factor was quantified using population attributable fractions (PAFs), which were estimated by GBD based on exposure distributions, relative risks, and theoretical minimum risk exposure levels. We extracted PAFs and corresponding 95% UI, which were derived from the 2.5th and 97.5th percentiles of 1,000 posterior draws.

### Statistical analysis

Joinpoint regression analysis was performed using Joinpoint Regression Program version 5.2.0.0 (20). The model was fitted on the logarithm of the ASR and allowed a maximum of five joinpoints. The optimal number of joinpoints was selected using Monte Carlo permutation tests with an overall significance level of 0.05. APC was calculated for each segment, and AAPC was estimated as a weighted average of APCs across the study period. The AAPC was calculated as follows:


AAPC = [∑i=1 k(APCi × Li)]/[∑i=1 kLi]

where APC_i_ represents the APC of the i-th segment, L_i_ denotes the length of the corresponding segment, and k is the total number of segments.

To explore short-term trend patterns around the COVID-19 pandemic, we conducted a segmented analysis comparing the AAPC during the pre-pandemic period (2010 to 2019) and the pandemic period (2019 to 2023). The year 2019 was used as the dividing point because it preceded the major disruption of health services caused by COVID-19 (21). A p-value less than 0.05 was considered statistically significant.

Statistical analyses were performed using R software (version 4.3.1), Joinpoint Regression Program (version 5.2.0.0), and JD_GBDR (version 2.37). Data processing was conducted using the tidyverse and data.table packages. Figures were generated using ggplot2, ggpubr, and patchwork. Joinpoint regression was applied to estimate temporal trends and calculate AAPCs. Decomposition analysis was performed using custom R scripts based on the Das Gupta method to quantify the contributions of population growth, population aging, and epidemiological changes to changes in pancreatic cancer burden.

## Results

### Temporal trends in the burden of pancreatic cancer in China, 1990–2023

**Figure 1:**
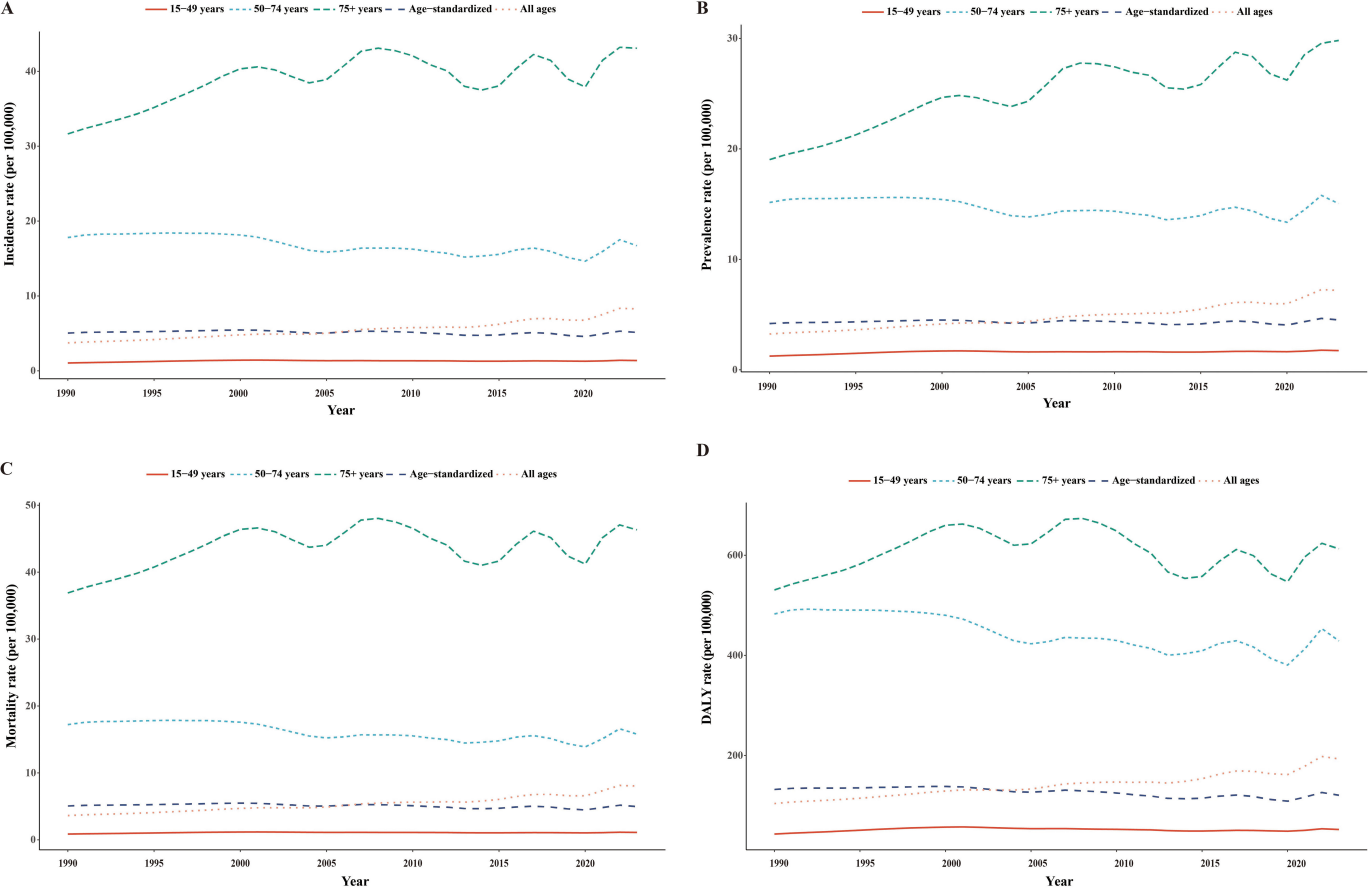
Trends in pancreatic cancer indicators in China by age group (1990-2023). Age-specific rates are shown for three age groups: 15–49 years, 50–74 years, and ≥75 years, with different colors representing different age groups. The age-standardized curve represents the all-age standardized rate. **(A)** Incidence rate; **(B)** prevalence rate; **(C)** mortality rate; **(D)** disability-adjusted life year (DALY) rate. All rates are expressed per 100,000 population.

From 1990 to 2023, the absolute burden of pancreatic cancer in China increased substantially, but most ASRs remained relatively stable. In 2023, China reported 118,381 incident cases (95% UI 103,773–135,339), 102,845 prevalent cases (95% UI 89,758–117,757), and 114,949 deaths (95% UI 101,214–131,619). The disease burden reached 2,765,626 DALYs (95% UI 2,455,494–3,137,032). Compared with 1990, the numbers of incident cases, prevalent cases, deaths, and DALYs increased by 169.18%, 169.00%, 168.92%, and 125.46%, respectively. Among the age-standardized indicators, only ASPR showed a statistically significant increase, rising from 4.19 to 4.51, with an AAPC of 0.38% (95% CI 0.06 to 0.70) (**Figure 1**; **Table 1**).

**Table 1:** Age-standardized rates and burden of pancreatic cancer in China, 1990-2023.

Indicator	Sex	Number (1990, 95%UI)	Number (2023, 95%UI)	Percent change (%)	ASR (1990, 95%UI)	ASR (2023, 95%UI)	AAPC of ASR (95% CI)
Death	Both	42731(36787,49928)	114949(101214,131619)	169.00	5.06(4.35,5.92)	4.97(4.37,5.70)	-0.14 (-0.65 to 0.38)
	Female	17569(14286,21859)	44882(36240,57512)	155.45	4.09(3.33,5.11)	3.69(2.99,4.69)	-0.48 (-0.79 to -0.16)*
	Male	25162(20574,32058)	70067(60025,82318)	178.46	6.11(4.98,7.81)	6.32(5.42,7.43)	0.22 (-0.32 to 0.76)
DALY	Both	1226645(1062371,1439160)	2765626(2455494,3137032)	125.46	132.16(114.24,154.80)	120.60(106.58,136.65)	-0.08 (-0.42 to 0.25)
	Female	478088(393469,584508)	984617(824039,1222325)	105.95	103.99(85.37,127.85)	82.47(69.13,101.35)	-0.58 (-0.89 to -0.26)*
	Male	748558(611242,941878)	1781009(1519033,2082466)	137.93	160.47(131.07,203.71)	159.13(136.06,184.47)	-0.02 (-0.32 to 0.28)
Incidence	Both	43978(37940,51580)	118381(103773,135339)	169.18	5.05(4.34,5.91)	5.13(4.48,5.88)	-0.04 (-0.54 to 0.47)
	Female	17841(14548,22131)	45701(37103,57589)	156.15	4.06(3.31,5.06)	3.78(3.07,4.72)	0.00 (-0.23 to 0.23)
	Male	26136(21374,33249)	72680(62030,85513)	178.08	6.09(4.96,7.80)	6.53(5.59,7.69)	0.33 (-0.19 to 0.85)
Prevalence	Both	38244(33099,44391)	102845(89758,117757)	168.92	4.19(3.63,4.87)	4.51(3.92,5.19)	0.38 (0.06 to 0.70)*
	Female	15184(12372,18223)	39398(32696,48491)	159.48	3.34(2.71,4.03)	3.33(2.77,4.08)	0.01 (-0.33 to 0.35)
	Male	23060(19072,28536)	63447(53846,72578)	175.14	5.05(4.17,6.25)	5.71(4.86,6.56)	0.48 (0.01 to 0.95)*

AAPC, average annual percentage change; ASR, age-standardized rate; CI, confidence interval; DALY, disability-adjusted life year; UI, uncertainty interval. The symbol * indicates p < 0.05. Trends with 95% CIs including 0 were considered statistically non-significant.

### Sex-specific trends

**Figure 2:**
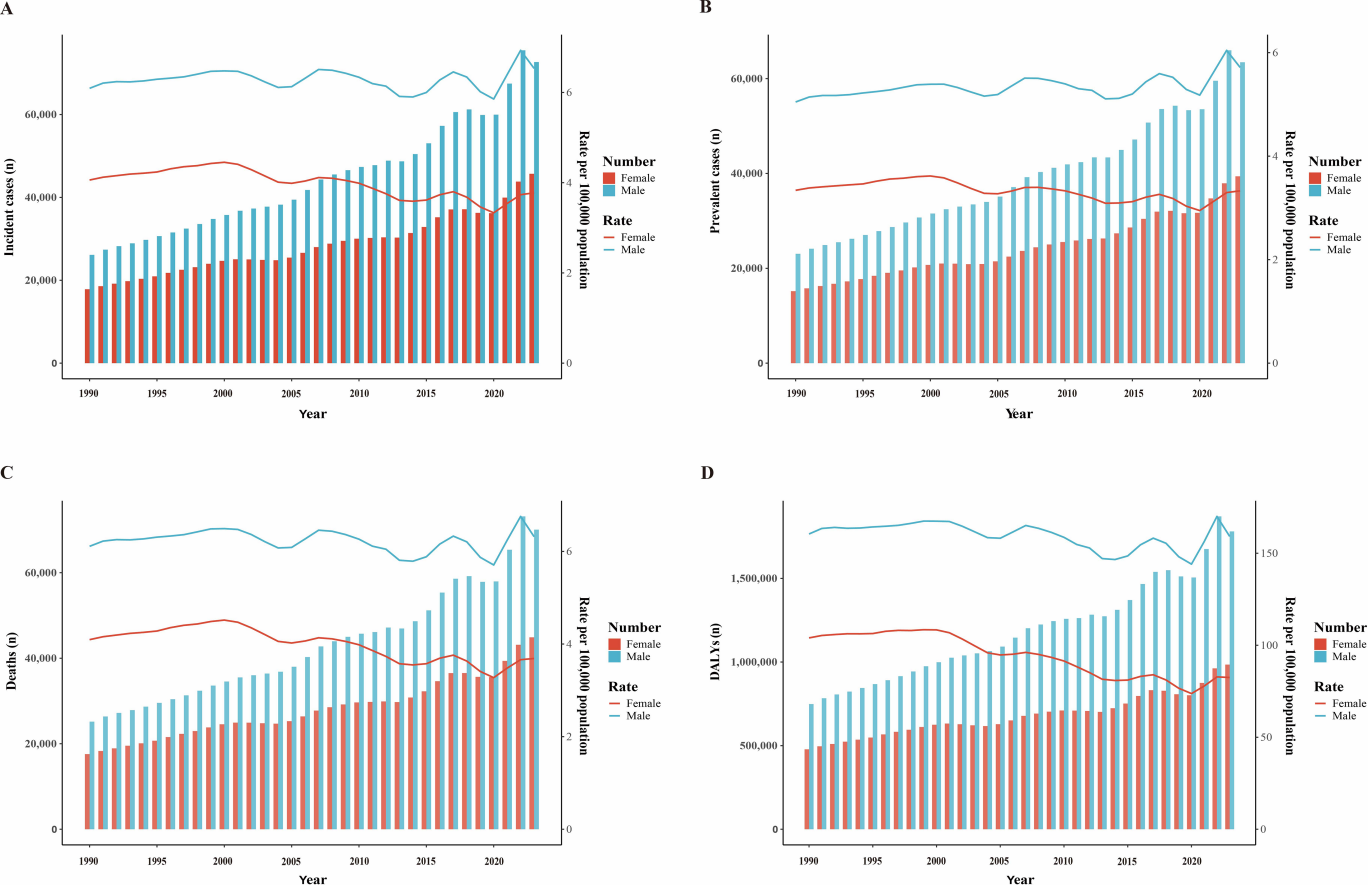
The disease burden of pancreatic cancer in China by sex, 1990–2023. The absolute numbers and age-standardized rates are presented for **(A)** incidence, **(B)** prevalence, **(C)** deaths, and **(D)** disability-adjusted life years (DALYs). Bars represent absolute numbers, and lines represent age-standardized rates per 100,000 population. Different colors indicate males and females.

The absolute burden of pancreatic cancer increased in both males and females in China from 1990 to 2023, and was consistently higher in males. In 2023, males had 72,680 incident cases (95% UI 62,030-85,513), 63,447 prevalent cases (95% UI 53,846-72,578), 70,067 deaths (95% UI 60,025-82,318), and 1,781,009 DALYs (95% UI 1,519,033-2,082,466), compared with 45,701 (95% UI 37,103-57,589), 39,398 (95% UI 32,696-48,491), 44,882 (95% UI 36,240-57,512), and 984,617 (95% UI 824,039-1,222,326) in females. Compared with 1990, the absolute numbers of incident cases, prevalent cases, deaths and DALYs in males increased by 178.08%, 175.14%, 178.46%, and 137.93%. In females, the corresponding increases were 156.15%, 159.48%, 155.45% and 105.95%. The ASIR was statistically stable in both sexes. The ASPR increased significantly in males, from 5.05 to 5.71, with an AAPC of 0.48% (95% CI 0.01 to 0.95), but it was stable in females. Among females, the ASMR declined from 4.09 to 3.69, with an AAPC of −0.48% (95% CI −0.79 to −0.16), and the ASDR declined from 103.99 to 82.47, with an AAPC of −0.58% (95% CI −0.89 to −0.26). The ASMR and ASDR in males showed no statistically significant long-term changes (**Figure 2**; **Table 1**).

### Age-specific trends

Pancreatic cancer burden varied across the 15–49, 50–74, and ≥75 years age groups from 1990 to 2023. The ≥75 years age group consistently had the highest burden. In this group, the incidence rate increased from 31.64 to 43.08, with an AAPC of 0.89% (95% CI 0.36 to 1.42). The incidence rate also increased in the 15–49 years age group, from 1.05 to 1.38, with an AAPC of 0.87% (95% CI 0.68 to 1.06). The incidence rate for the 50–74 years age group remained relatively stable. Prevalence rosed most rapidly in the ≥75 years group. The prevalence rate rose from 19.03 to 29.81, with an AAPC of 1.33% (95% CI 0.83 to 1.83), and the number of prevalent cases increased by 524.14% from 1990 to 2023. The 15–49 years age group also showed a significant increase in the prevalence rate, with an AAPC of 1.01% (95% CI 0.84 to 1.19), but the rate for the 50–74 years age group remained stable. The mortality rate in the ≥75 years age group reached 46.32 in 2023, with an AAPC of 0.73% (95% CI 0.08 to 1.39), and the absolute number of deaths increased by 400.30%. A significant rise was also observed in the 15–49 years age group, with an AAPC of 0.78% (95% CI 0.57 to 1.00). The mortality rate for the 50–74 years age group declined from 17.22 to 15.79, with no statistically significant long-term trend. The DALY rate was highest in the ≥75-year age group across the study period (**Figure 1**; **Table 2**).

**Table 2:** Age-specific trends in pancreatic cancer burden in China, 1990-2023.

Metric	Measure	Number (1990, 95%UI)	Number (2023, 95%UI)	Percent change (%)	Rate (1990, 95%UI)	Rate (2023, 95%UI)	AAPC of rate (95% CI)
Death	15–49 years	5753(4889,6826)	7141(5788,8615)	24.12	0.86(0.73,1.02)	1.11(0.90,1.33)	0.78 (0.57 to 1.00)*
	50–74 years	29804(25518,34997)	71914(62671,83657)	141.29	17.22(14.74,20.22)	15.79(13.76,18.37)	-0.08 (-0.51 to 0.35)
	75+ years	7174(6126,8659)	35894(28788,43870)	400.30	36.89(31.50,44.52)	46.32(37.15,56.61)	0.73 (0.08 to 1.39)*
	All ages	42731(36787,49928)	114949(101214,131619)	169.00	3.62(3.12,4.23)	8.03(7.07,9.20)	2.48 (2.09 to 2.88)*
DALY	15–49 years	288235(246164,340716)	337145(273693,406208)	16.97	43.11(36.81,50.96)	52.22(42.39,62.92)	0.53 (0.37 to 0.69)*
	50–74 years	835216(715321,983128)	1953579(1689016,2245121)	133.90	482.57(413.30,568.03)	428.97(370.87,492.98)	-0.51 (-0.77 to -0.25)*
	75+ years	103195(88413,124624)	474902(384354,576315)	360.20	530.59(454.59,640.77)	612.82(495.98,743.69)	0.42 (-0.10 to 0.93)
	All ages	1226645(1062371,1439160)	2765626(2455494,3137032)	125.46	104.03(90.10,122.05)	193.31(171.64,219.27)	1.90 (1.52 to 2.29)*
Incidence	15–49 years	7018(5958,8274)	8900(7220,10709)	26.82	1.05(0.89,1.24)	1.38(1.12,1.66)	0.87 (0.68 to 1.06)*
	50–74 years	30806(26338,36178)	76096(65832,87604)	147.01	17.80(15.22,20.90)	16.71(14.46,19.24)	-0.16 (-0.61 to 0.30)
	75+ years	6154(5257,7454)	33385(26343,41532)	442.52	31.64(27.03,38.33)	43.08(33.99,53.59)	0.89 (0.36 to 1.42)*
	All ages	43978(37940,51580)	118381(103773,135339)	169.18	3.73(3.22,4.37)	8.27(7.25,9.46)	2.32 (2.05 to 2.58)*
Prevalence	15–49 years	8311(7091,9942)	11247(9481,12976)	35.32	1.24(1.06,1.49)	1.74(1.47,2.01)	1.01 (0.84 to 1.19)*
	50–74 years	26231(22773,30467)	68494(60946,77831)	161.12	15.16(13.16,17.60)	15.04(13.38,17.09)	0.04 (-0.39 to 0.47)
	75+ years	3702(3092,4428)	23104(18527,28573)	524.14	19.03(15.90,22.77)	29.81(23.91,36.87)	1.33 (0.83 to 1.83)*
	All ages	38244(33099,44391)	102845(89758,117757)	168.92	3.24(2.81,3.76)	7.19(6.27,8.23)	2.38 (2.16 to 2.60)*

AAPC, average annual percentage change; CI, confidence interval; UI, uncertainty interval. The symbol * indicates p < 0.05. Trends with 95% CIs including 0 were considered statistically non-significant.

### Decomposition analysis

**Figure 3:**
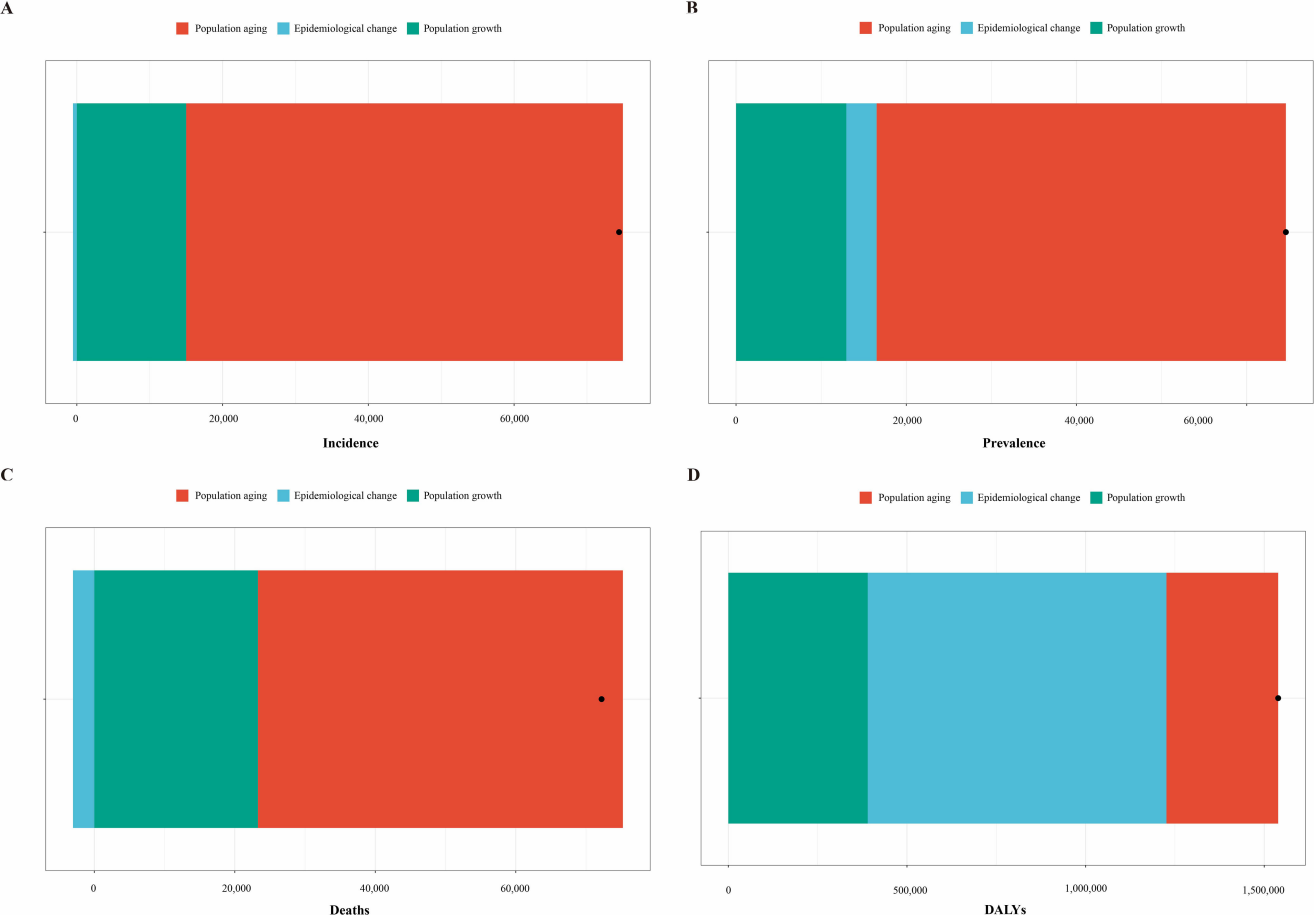
Decomposition of changes in pancreatic cancer burden in China from 1990 to 2023. The total change in **(A)** incident cases, **(B)** prevalent cases, **(C)** deaths, and **(D)** DALYs was decomposed into contributions from population growth, population aging, and epidemiological change.

Decomposition analysis showed that the increases in deaths, incidence, and prevalence were mainly driven by population aging, followed by population growth. Epidemiological change had a limited effect on incidence, deaths, or prevalence. But the increase in DALYs was due mainly to epidemiological change, with additional contributions from population growth and a smaller effect of aging. In this analysis, epidemiological change refers to changes in age-specific rates after accounting for population size and age structure (**Figure 3**).

### Risk-attributable burden

**Figure 4:**
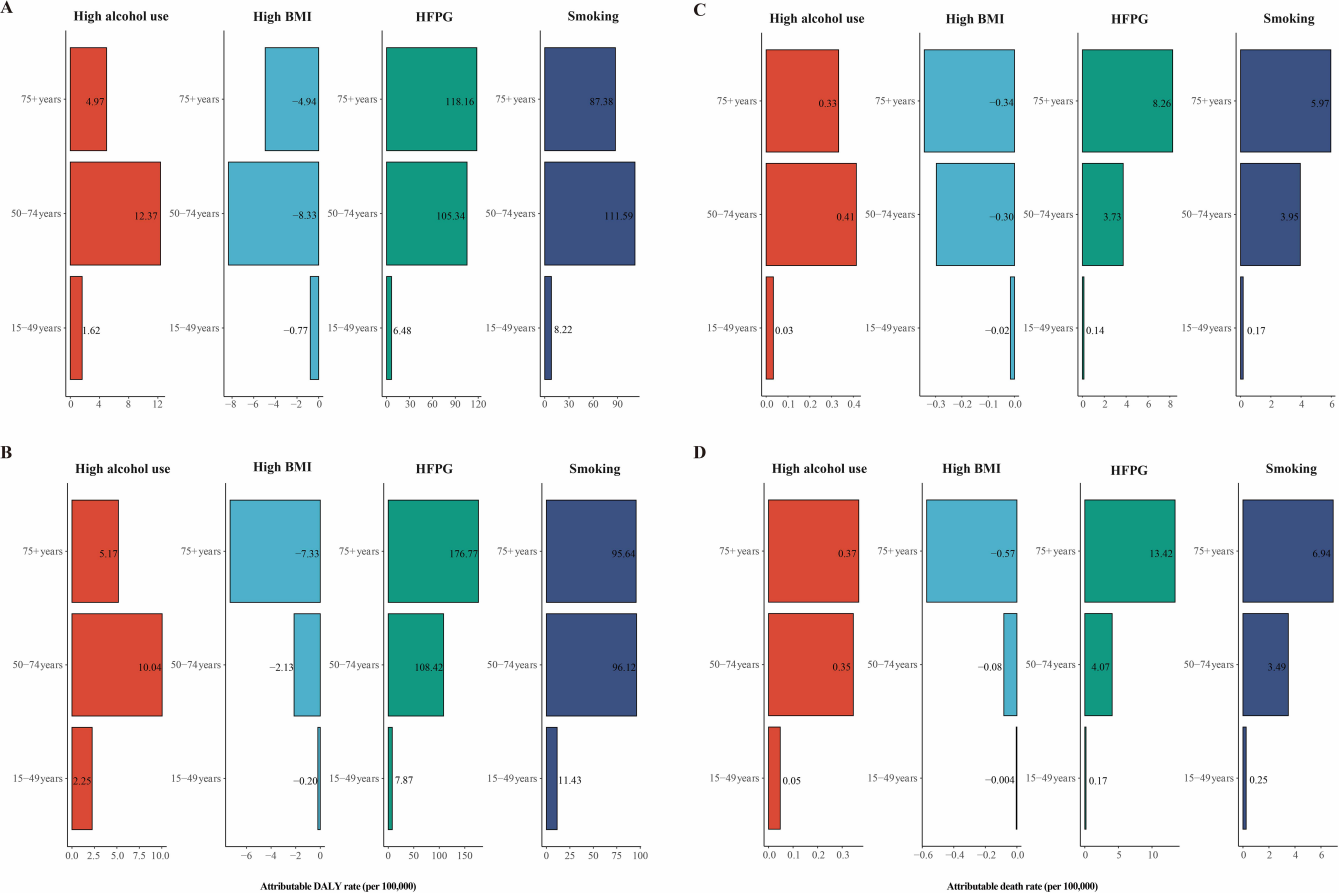
Age-specific burden of pancreatic cancer attributable to major risk factors in China, 1990 and 2023. Attributable death rates in **(A)** 1990 and **(B)** 2023; attributable DALY rates in **(C)** 1990 and **(D)** 2023. Rates are expressed per 100,000 population. The assessed risk factors included smoking, high fasting plasma glucose, high body-mass index, and high alcohol use. BMI, body-mass index; HFPG, high fasting plasma glucose.

**Figure 5:**
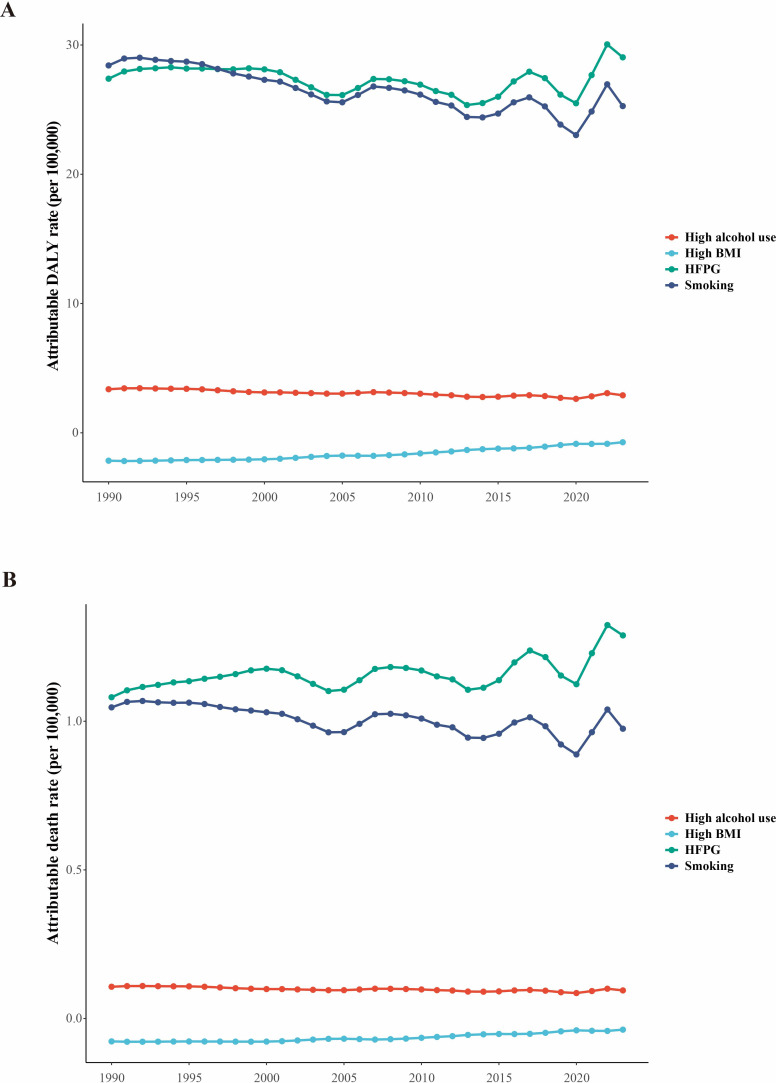
Temporal trends in the burden of pancreatic cancer attributable to major factors in China, 1990–2023. Age-standardized rates attributable to major risk factors are displayed as separate curves, with different colors indicating different risk factors. The assessed risk factors include smoking, high fasting plasma glucose, high body-mass index, and high alcohol use. **(A)** Age-standardized disability-adjusted life year (DALY) rates. **(B)** Age-standardized mortality rates. All rates are expressed per 100,000 population. BMI, body-mass index; HFPG, high fasting plasma glucose.

From 1990 to 2023, the risk-attributable burden of pancreatic cancer showed different patterns across the four assessed risk factors. For HFPG, the attributable ASMR increased from 1.08 to 1.29 per 100,000, with an AAPC of 0.39% (95% CI 0.14 to 0.65). The attributable ASMR and ASDR for high alcohol use decreased significantly, with AAPCs of −0.56% (95% CI -0.78 to -0.33) and −0.30% (95% CI -0.61 to -0.00), respectively. Trends for smoking were not statistically significant. In 2023, HFPG accounted for higher attributable deaths and DALYs than smoking, particularly in the ≥75 years age group (**Figures 4**, **5**; **Table 3**).

**Table 3:** Burden of pancreatic cancer attributable to risk factors in China, 1990-2023.

Measure	Risk factors	Number (1990, 95%UI)	Number (2023, 95%UI)	Percent change (%)	ASR (1990, 95%UI)	ASR (2023, 95%UI)	AAPC of ASR (95% CI)
Deaths	Smoking	9148(7187,11413)	22860(18949,27858)	151.14	1.05(0.82,1.30)	0.97(0.81,1.19)	-0.12 (-0.71 to 0.47)
	High alcohol use	1002(147,1667)	2165(418,3430)	116.87	0.11(0.02,0.18)	0.09(0.02,0.15)	-0.56 (-0.78 to -0.33)*
	High body-mass index	-684(-2112,5)	-847(-5177,1637)	23.54	-0.08(-0.23,0.00)	-0.04(-0.22,0.07)	NA
	High fasting plasma glucose	8966(5999,12897)	30021(23516,38383)	236.45	1.08(0.74,1.55)	1.29(1.01,1.65)	0.39 (0.14 to 0.65)*
DALYs	Smoking	265123(209110,335055)	585652(484319,708327)	121.88	28.42(22.40,35.77)	25.28(20.92,30.58)	-0.28 (-0.75 to 0.18)
	High alcohol use	33208(4595,56053)	64270(11840,103410)	94.14	3.37(0.48,5.66)	2.90(0.52,4.69)	-0.30 (-0.61 to -0.00)*
	High body-mass index	-20544(-64214,285)	-16683(-121572,46075)	-19.01	-2.16(-6.69,0.02)	-0.73(-5.35,1.99)	NA
	High fasting plasma glucose	248664(161199,367206)	681555(537021,869608)	175.29	27.39(18.06,39.77)	29.05(22.83,37.12)	0.03 (-0.21 to 0.26)

AAPC, average annual percentage change; ASR, age-standardized rate; CI, confidence interval; DALY, disability-adjusted life year; UI, uncertainty interval. The symbol * indicates p < 0.05. Trends with 95% CIs including 0 were considered statistically non-significant.

### Pandemic-era sensitivity analysis

The pandemic-era sensitivity analysis compared AAPCs for 2010–2019 and 2019–2023 (**Table 4**). From 2010 to 2019, ASIR, ASMR, and ASDR all declined significantly, with AAPCs of −0.84% (95% CI −1.40 to −0.29), −0.98% (95% CI −1.55 to −0.41), and −1.09% (95% CI −1.59 to −0.58). During 2019–2023, the AAPC point estimates for ASIR, ASMR, and ASDR were positive, at 1.52% (95% CI −7.99 to 11.04), 1.18% (95% CI −8.35 to 10.71), and 1.04% (95% CI −8.57 to 10.65). However, all three confidence intervals were wide and included 0. These results indicate substantial statistical uncertainty in the short-term 2019–2023 trend estimates (**Table 4**).

**Table 4:** Average annual percentage changes in age-standardized pancreatic cancer rates before and after the COVID-19 pandemic, China.

Measure	Period	AAPC
ASMR	2010–2019	-0.98 (-1.55 to -0.41)*
	2019–2023	1.18 (-8.35 to 10.71)
ASDR	2010–2019	-1.09 (-1.59 to -0.58)*
	2019–2023	1.04 (-8.57 to 10.65)
ASIR	2010–2019	-0.84 (-1.40 to -0.29)*
	2019–2023	1.52 (-7.99 to 11.04)
ASPR	2010–2019	-0.47 (-0.98 to 0.05)
	2019–2023	1.45 (-6.87 to 9.77)

AAPC, average annual percentage change. The symbol * indicates p < 0.05. Trends with 95% CIs including 0 were considered statistically non-significant.

## Discussion

Using the latest GBD 2023 data, we systematically evaluated the disease burden of pancreatic cancer in China from 1990 to 2023. The main finding was that the absolute burden increased markedly, while most ASRs remained generally stable. This suggests that the rising burden of pancreatic cancer in China was mainly reflected in the growing number of affected people, rather than increases in ASRs.

From a global perspective, China’s epidemiologic profile is rapidly converging toward that of high-Socio-demographic Index countries. Previous global assessments indicate that the highest pancreatic cancer incidence has been concentrated in highly industrialized regions, such as Eastern Europe and North America. Our data suggest that China is quickly narrowing this gap (22, 23). Compared with several low-incidence regions, including parts of sub-Saharan Africa and Southeast Asia, China has seen a substantial increase in the absolute burden of pancreatic cancer amid rapid urbanization, population aging, and lifestyle changes (24, 25).

The higher burden in males may reflect more than differences in smoking and alcohol exposure. Biological factors, including sex hormone-related pathways, fat distribution, glucose metabolism, and inflammatory responses, may also contribute to sex-specific differences in pancreatic cancer risk (26–28). In addition, men may delay seeking medical care for vague early symptoms or participate less in routine health examinations, which could affect the timing of diagnosis (29, 30).

The age-specific findings also deserve attention. The ≥75 years age group consistently had the highest burden of pancreatic cancer in China. Given the demographic transition in China, the expansion of the population aged over 75 may further increase the absolute burden of this malignant tumor in the coming decades (15, 31). Recent studies have reported increasing early-onset gastrointestinal cancers worldwide, suggesting that changes in obesity, diabetes, diet, physical activity, and other lifestyle-related factors may be involved (32, 33). For pancreatic cancer, early symptoms are often nonspecific, and clinicians may have lower suspicion in younger adults, which may delay diagnosis. For younger adults, these findings support improved clinical awareness and risk-based evaluation, particularly among individuals with persistent abdominal symptoms, new-onset diabetes, family history, or other high-risk features (32, 34). Given the insidious onset of pancreatic cancer, AI-assisted tools may complement clinical assessment by supporting risk stratification and diagnostic decision-making in patients with atypical or early-onset presentations (35–38).

Decomposition analysis indicated that population aging was the main contributor to the increases in incident cases, deaths, and prevalent cases. For DALYs, the larger contribution of epidemiological change may reflect changes in age-specific disease burden after accounting for population size and age structure. This is because DALYs are influenced not only by the number of deaths, but also by age at death and disability burden. As the elderly population continues to expand in China, the absolute number of pancreatic cancer cases is likely to remain a major challenge for the health system (31).

Regarding risk factors, HFPG showed the largest attributable burden among the four modifiable risk factors assessed in this study. In 2023, HFPG accounted for more attributable deaths and DALYs than smoking, and its ASMR was also higher than that of smoking. This finding suggests that metabolic risk may be playing an increasingly important role in the pancreatic cancer burden in China. The growing contribution of HFPG is biologically plausible. Long-term diabetes and hyperglycemia have been associated with increased pancreatic cancer risk, possibly through insulin resistance, hyperinsulinemia, chronic inflammation, oxidative stress, and changes in the pancreatic microenvironment (15, 39, 40). At the population level, rapid aging, dietary changes, reduced physical activity, and the increasing prevalence of diabetes may further increase the impact of metabolic risk on pancreatic cancer burden in China. These findings suggest that pancreatic cancer prevention should include metabolic risk management, such as better detection and control of diabetes, promotion of healthy diets and physical activity, together with continued tobacco control (41, 42). However, these results should be interpreted within the GBD comparative risk assessment framework and do not indicate that HFPG is the leading risk factor among all possible causes of pancreatic cancer (16). Negative attributable estimates for high body-mass index were retained as extracted from GBD 2023. These values may occur when the modeled exposure distribution is below the theoretical minimum risk exposure level for a specific risk–outcome pair and should therefore be interpreted cautiously (43, 44).

The pandemic-era sensitivity analysis suggested a possible change in the direction of pancreatic cancer trends after 2019. In our analysis, ASIR, ASMR, and ASDR declined significantly during 2010–2019, whereas their AAPC point estimates were positive during 2019–2023. However, the 95% CIs for the post-2019 estimates were wide and included 0, indicating that these short-term changes were not statistically significant. Therefore, the observed changes should be interpreted as possible short-term fluctuations rather than definitive trend reversals. Previous studies have reported that the COVID-19 pandemic disrupted cancer screening, diagnostic testing, endoscopy, surgery, and cancer treatment services (11, 45, 46). For pancreatic cancer, delayed imaging, endoscopy, specialist referral, or surgery may be clinically important because the disease often has a narrow window for curative treatment (34, 47). These findings suggest that pandemic-related healthcare disruption may have contributed to short-term changes in incidence and mortality estimates, but longer follow-up and validation using cancer registry data are needed.

This study is subject to limitations inherent to the GBD methodology (13, 14). Estimates may be biased in settings with sparse high-quality cancer registry data, despite the inclusion of best available evidence. Data aggregation precluded the distinction between specific histological subtypes, such as pancreatic ductal adenocarcinoma and neuroendocrine tumors. Furthermore, this study was conducted at the national level and could not assess provincial or regional heterogeneity. Differences in population aging, risk exposure, healthcare access, cancer registry coverage, and diagnostic capacity may contribute to regional variation in pancreatic cancer burden across China. National estimates may therefore mask local high-burden areas. Future studies using provincial data are needed to support more targeted prevention strategies and resource allocation.

## Conclusion

Using the most recent GBD 2023 estimates, this study systematically characterized pancreatic cancer burden trends in China from 1990 to 2023, covering incidence, mortality, prevalence, and DALYs. Despite relatively stable ASRs, the absolute numbers of incident cases, deaths, and prevalent cases increased substantially, largely reflecting population aging. The burden remained higher in males and increased sharply with age. Risk-attributable analysis suggested an increasing contribution of metabolic risk, with HFPG accounting for the largest attributable burden among the four assessed GBD risk factors in 2023. Given the model-dependent nature of GBD estimates, these findings warrant validation against data from the Chinese Cancer Registry and national surveillance systems. Future prevention strategies should integrate metabolic risk management with established tobacco control measures to address the dual challenges of an aging population and an evolving risk landscape.

## Data Availability

The raw data supporting the conclusions of this article will be made available by the authors, without undue reservation.

## Author's Note

Hao Wu, the corresponding author (hwu25@mails.jlu.edu.cn), is a GBD Collaborator and led the research.

## References

[1] Halbrook CJ Lyssiotis CA Pasca Di Magliano M Maitra A . Pancreatic cancer: advances and challenges. Cell. (2023) 186:1729–54. doi: 10.1016/j.cell.2023.02.014 37059070 PMC10182830

[2] Stoffel EM Brand RE Goggins M . Pancreatic cancer: changing epidemiology and new approaches to risk assessment, early detection, and prevention. Gastroenterology. (2023) 164:752–65. doi: 10.1053/j.gastro.2023.02.012 36804602 PMC10243302

[3] Huang L Wang Z Wang H Zhao L Jiang H Zhang B . Nutrition transition and related health challenges over decades in China. Eur J Clin Nutr. (2021) 75:247–52. doi: 10.1038/s41430-020-0674-8 32620907

[4] Gong P Liang S Carlton EJ Jiang Q Wu J Wang L . Urbanisation and health in China. Lancet. (2012) 379:843–52. doi: 10.1016/S0140-6736(11)61878-3 22386037 PMC3733467

[5] Pan XF Wang L Pan A . Epidemiology and determinants of obesity in China. Lancet Diabetes Endocrinol. (2021) 9:373–92. doi: 10.1016/S2213-8587(21)00045-0 34022156

[6] Xu Y Lu J Li M Wang T Wang K Cao Q . Diabetes in China part 1: epidemiology and risk factors. Lancet Public Health. (2024) 9:e1089–97. doi: 10.1016/S2468-2667(24)00250-0 39579774

[7] Chen J Chen H Zhang T Yin X Man J Yang X . Burden of pancreatic cancer along with attributable risk factors in China from 1990 to 2019, and projections until 2030. Pancreatology. (2022) 22:608–18. doi: 10.1016/j.pan.2022.04.011 35513974

[8] Kan C Liu N Zhang K Wu D Liang Y Cai W . Global, regional, and national burden of pancreatic cancer, 1990-2019: results from the Global Burden of Disease Study 2019. Ann Glob Health. (2023) 89:33. doi: 10.5334/aogh.4019 37252335 PMC10215993

[9] Du R Wang Y Pan M Zhu J Zhao Y Zhang C . Global, regional, and national burdens of pancreatic cancer attributable to smoking from 1990 to 2021 and the projections to 2035: a systematic analysis from the global burden of disease study 2021. Front Oncol. (2025) 15:1547029. doi: 10.3389/fonc.2025.1547029 40519307 PMC12163048

[10] GBD 2017 Pancreatic Cancer Collaborators . The global, regional, and national burden of pancreatic cancer and its attributable risk factors in 195 countries and territories, 1990-2017: a systematic analysis for the Global Burden of Disease Study 2017. Lancet Gastroenterol Hepatol. (2019) 4:934–47. doi: 10.1016/S2468-1253(19)30347-4 31648972 PMC7026711

[11] Teglia F Angelini M Astolfi L Casolari G Boffetta P . Global association of COVID-19 pandemic measures with cancer screening: a systematic review and meta-analysis. JAMA Oncol. (2022) 8:1287–93. doi: 10.1001/jamaoncol.2022.2617 35797056 PMC9264214

[12] Jones CM Radhakrishna G Aitken K Bridgewater J Corrie P Eatock M . Considerations for the treatment of pancreatic cancer during the COVID-19 pandemic: the UK consensus position. Br J Cancer. (2020) 123:709–13. doi: 10.1038/s41416-020-0980-x 32641867 PMC7341025

[13] GBD 2023 Causes of Death Collaborators . Global burden of 292 causes of death in 204 countries and territories and 660 subnational locations, 1990-2023: a systematic analysis for the Global Burden of Disease Study 2023. Lancet. (2025) 406:1811–72. doi: 10.1016/S0140-6736(25)01917-8 41092928 PMC12535838

[14] Wu Y Yang G Zengin G Ren S Li M . Trends in gallbladder and biliary tract cancer global burden (1990–2021): a socioeconomic gradient analysis and projections for 2040. BIOI. (2026) 7:1–17. doi: 10.15212/bioi-2025-0174

[15] Huang J Lok V Ngai CH Zhang L Yuan J Lao XQ . Worldwide burden of, risk factors for, and trends in pancreatic cancer. Gastroenterology. (2021) 160:744–54. doi: 10.1053/j.gastro.2020.10.007 33058868

[16] Brauer M Roth GA Aravkin AY Zheng P Abate KH Abate YH . Global burden and strength of evidence for 88 risk factors in 204 countries and 811 subnational locations, 1990–2021: a systematic analysis for the Global Burden of Disease Study 2021. Lancet. (2024) 403:2162–203. doi: 10.1016/S0140-6736(24)00933-4 38762324 PMC11120204

[17] Zhu J Li S Li X Wang L Du L Qiu Y . Impact of population ageing on cancer-related disability-adjusted life years: a global decomposition analysis. J Glob Health. (2024) 14:4144. doi: 10.7189/jogh.14.04144 39024622 PMC11259023

[18] Gao N Yang N Huang J . Decomposition and forecasting of colorectal cancer burden attributable to high body mass index and high fasting plasma glucose, 1990–2021: a GBD 2021 study. Front Nutr. (2025) 12:1652676. doi: 10.3389/fnut.2025.1652676 41479670 PMC12753452

[19] Li J Gao Z Bai H Wang W Li Y Lian J . Global, regional, and national total burden related to hepatitis B in children and adolescents from 1990 to 2021. BMC Public Health. (2024) 24:2936. doi: 10.1186/s12889-024-20462-4 39443929 PMC11515762

[20] Yang P Huang W Xu Y Teng Y Shu P . Trends and projections of the burden of gastric cancer in China and G20 countries: a comparative study based on the global burden of disease database 2021. Int J Surg. (2025) 111:4854–65. doi: 10.1097/JS9.0000000000002464 40359560

[21] Wang S Lv J Wang L Wu H Cao X . Colorectal cancer in China, 1990 to 2023: trends, modifiable risks, and prevention priorities based on Global Burden of Disease 2023 estimates. J Gastrointest Canc. (2026) 57:21. doi: 10.1007/s12029-026-01400-6 41557031

[22] Zhou M Wang H Zeng X Yin P Zhu J Chen W . Mortality, morbidity, and risk factors in China and its provinces, 1990–2017: a systematic analysis for the Global Burden of Disease Study 2017. Lancet. (2019) 394:1145–58. doi: 10.1016/S0140-6736(19)30427-1 31248666 PMC6891889

[23] Sung H Ferlay J Siegel RL Laversanne M Soerjomataram I Jemal A . Global cancer statistics 2020: GLOBOCAN estimates of incidence and mortality worldwide for 36 cancers in 185 countries. CA A Cancer J Clin. (2021) 71:209–49. doi: 10.3322/caac.21660 33538338

[24] Liu C Liu P Liu X Niu W Wu P Yu J . Global, regional, and national burden and trends of pancreatic cancer, 1990–2021: a systematic analysis for the Global Burden of Disease Study 2021. Front Oncol. (2025) 15:1671856. doi: 10.3389/fonc.2025.1671856 41256325 PMC12620227

[25] Koh YX Zhao Y Thien A Guo Y Tan HL Chua DW . Pancreatic cancer burden across Southeast Asia from 1990 to 2021: an analysis of incidence and mortality based on the global burden of disease study 2021. Pancreatology. (2026) 26:83–94. doi: 10.1016/j.pan.2025.09.031 41102102

[26] Zhang M Yang L Wang L Jiang Y Huang Z Zhao Z . Trends in smoking prevalence in urban and rural China, 2007 to 2018: findings from 5 consecutive nationally representative cross-sectional surveys. PloS Med. (2022) 19:e1004064. doi: 10.1371/journal.pmed.1004064 36006870 PMC9409540

[27] Wang M Gorelick F Bhargava A . Sex differences in the exocrine pancreas and associated diseases. Cell Mol Gastroenterol Hepatol. (2021) 12:427–41. doi: 10.1016/j.jcmgh.2021.04.005 33895424 PMC8255941

[28] Grahovac J Đurić A Tanić M Krivokuća A . Sex-related differences in pancreatic ductal adenocarcinoma progression and response to therapy. IJMS. (2024) 25:12669. doi: 10.3390/ijms252312669 39684385 PMC11641295

[29] Fish JA Prichard I Ettridge K Grunfeld EA Wilson C . Psychosocial factors that influence men’s help‐seeking for cancer symptoms: a systematic synthesis of mixed methods research. Psycho-Oncology. (2015) 24:1222–32. doi: 10.1002/pon.3912 26202128

[30] Davis JL Buchanan KL Katz RV Green BL . Gender differences in cancer screening beliefs, behaviors, and willingness to participate: implications for health promotion. Am J Mens Health. (2012) 6:211–7. doi: 10.1177/1557988311425853 22071507 PMC3776317

[31] Gong J Wang G Wang Y Chen X Chen Y Meng Q . Nowcasting and forecasting the care needs of the older population in China: analysis of data from the China Health and Retirement Longitudinal Study (CHARLS). Lancet Public Health. (2022) 7:e1005–13. doi: 10.1016/S2468-2667(22)00203-1 36423656 PMC9741660

[32] Jayakrishnan T Ng K . Early-onset gastrointestinal cancers: a review. JAMA. (2025) 334:1373–85. doi: 10.1001/jama.2025.10218 40674064 PMC13281743

[33] Danpanichkul P Suparan K Auttapracha T Tothanarungroj P Kongarin S Rakwong K . Early-onset gastrointestinal cancers and metabolic risk factors: global trends from the Global Burden of Disease Study 2021. Mayo Clin Proc. (2025) 100:1159–71. doi: 10.1016/j.mayocp.2024.10.021 39945699

[34] Park W Chawla A O’Reilly EM . Pancreatic cancer: a review. JAMA. (2021) 326:851–62. doi: 10.1001/jama.2021.13027 34547082 PMC9363152

[35] Ding H Feng Y Huang X Xu J Zhang T Liang Y . Deep learning‐based classification and spatial prognosis risk score on whole‐slide images of lung adenocarcinoma. Histopathology. (2023) 83:211–28. doi: 10.1111/his.14918 37071058

[36] Ding H Xia W Zhou Y Wei L Feng Y Wang Z . Evaluation and practical application of prompt-driven ChatGPTs for EMR generation. NPJ Digit Med. (2025) 8:77. doi: 10.1038/s41746-025-01472-x 39894840 PMC11788423

[37] Ding H Xia W Zhang L Mao Q Cao B Zhao Y . CT-based deep learning model for invasiveness classification and micropapillary pattern prediction within lung adenocarcinoma. Front Oncol. (2020) 10:1186. doi: 10.3389/fonc.2020.01186 32775302 PMC7388896

[38] Cao K Xia Y Yao J Han X Lambert L Zhang T . Large-scale pancreatic cancer detection via non-contrast CT and deep learning. Nat Med. (2023) 29:3033–43. doi: 10.1038/s41591-023-02640-w 37985692 PMC10719100

[39] Verhoeff K Shapiro AMJ . The bidirectional link between diabetes and pancreatic cancer: a diagnostic aid, risk factor, and potential target for future therapy. Mol Aspects Med. (2025) 106:101414. doi: 10.1016/j.mam.2025.101414 41005205

[40] Wu Y Sun R Ren S Zengin G Li M . Neuronal reshaping of the tumor microenvironment in tumorigenesis and metastasis: bench to clinic. Med Adv. (2025) 3:364–71. doi: 10.1002/med4.70044 41531421

[41] Wu Y Sun R Ren S Zengin G Li M . Tumor organoids: breakthroughs in clinical decision making, drug development, and translational advances beyond conventional models. Med Res. (2026) 2:26–40. doi: 10.1002/mdr2.70048 41531421

[42] Wu Y Sun R Liang J Zengin G Li M-Y . Food and medicine homology substances in cancer management. Food Sci Hum Wellness. (2026) 16:9251048. doi: 10.26599/FSHW.2026.9251048

[43] Hay SI Ong KL Santomauro DF A B Aalipour MA Aalruz H . Burden of 375 diseases and injuries, risk-attributable burden of 88 risk factors, and healthy life expectancy in 204 countries and territories, including 660 subnational locations, 1990–2023: a systematic analysis for the Global Burden of Disease Study 2023. Lancet. (2025) 406:1873–922. doi: 10.1016/S0140-6736(25)01637-X 41092926 PMC12535840

[44] Mansournia MA Altman DG . Population attributable fraction. BMJ. (2018) 360:k757. doi: 10.1136/bmj.k757 29472187

[45] Richards M Anderson M Carter P Ebert BL Mossialos E . The impact of the COVID-19 pandemic on cancer care. Nat Cancer. (2020) 1:565–7. doi: 10.1038/s43018-020-0074-y 35121972 PMC7238956

[46] Maringe C Spicer J Morris M Purushotham A Nolte E Sullivan R . The impact of the COVID-19 pandemic on cancer deaths due to delays in diagnosis in England, UK: a national, population-based, modelling study. Lancet Oncol. (2020) 21:1023–34. doi: 10.1016/S1470-2045(20)30388-0 32702310 PMC7417808

[47] Cai J Chen H Lu M Zhang Y Lu B You L . Advances in the epidemiology of pancreatic cancer: trends, risk factors, screening, and prognosis. Cancer Lett. (2021) 520:1–11. doi: 10.1016/j.canlet.2021.06.027 34216688

